# Assessing Pediatric Endodontic Referrals to University-Based Clinics: A Retrospective Chart Review

**DOI:** 10.3390/dj13110511

**Published:** 2025-11-03

**Authors:** Alice P. Chen, Civon Gewelber, Helpis Youssef, Jacob Marx, Man Hung

**Affiliations:** 1 College of Dental Medicine AEGD Program, Roseman University of Health Sciences College of Dental Medicine, Henderson, NV 89014, USA; 2 College of Dental Medicine DMD Program, Roseman University of Health Sciences College of Dental Medicine, 10894 S. River Front Parkway, South Jordan, UT 84095, USA; 3 Department of Family and Preventive Medicine, University of Utah, Salt Lake City, UT 84108, USA; 4 Huntsman Cancer Institute, Salt Lake City, UT 84112, USA

**Keywords:** pediatric dentistry, medicaid, root canal therapy, vital pulp therapy, endodontic referrals

## Abstract

**Background:** Timely dental care is essential to prevent complications and preserve natural teeth, yet inefficient referral practices, low reimbursement rates and systemic barriers continue to disproportionately affect Medicaid-enrolled children. This study assessed the appropriateness of root canal therapy (RCT) referrals to a University-based Advanced Education in General Dentistry (AEGD) program and examined diagnostic and treatment characteristics of referred cases. **Methods:** A retrospective review was conducted of pediatric patients aged 6–17 years who were referred by external dental providers to two AEGD clinics in Nevada, United States, between February and August 2024 for endodontic evaluation of carious permanent teeth. Demographic, tooth-type, pulp-status, and treatment outcome data were analyzed using descriptive statistics, chi-square tests, Kruskal–Wallis comparisons, and multivariable logistic regression models. **Results:** Among 154 referred patients, 96.8% (n = 149) were Medicaid beneficiaries. A total of 247 teeth were evaluated; 74.1% were molars. Pulp testing showed that 41.3% had healthy pulps and 16.6% had reversible pulpitis, while only 37.2% of teeth required RCT. Age differed significantly across pulp diagnoses (*p* = 0.0012), and older age independently predicted appropriate referral (adjusted OR = 1.18, 95% CI 1.07–1.31). Gender was not associated with follow-up compliance (*p* = 0.47). By November 2024, 53.4% of referred teeth had completed treatment, and 9.3% had no follow-up. **Conclusions:** More than half of the referral teeth did not require RCT, indicating a high rate of potentially avoidable referrals and highlighting gaps in diagnostic confidence and decision-making at the primary care level. **Practical Implications:** Enhancing diagnostic training and decision support for general dentists, particularly in vital pulp therapy for young permanent teeth, along with improving Medicaid reimbursement and standardizing referral protocols, may reduce inappropriate specialty referrals. In combination with broader policy reforms, these measures can improve system efficiency and expand access to timely, equitable pediatric dental care.

## 1. Introduction

Timely, appropriate dental care is a cornerstone of overall health and quality of life, yet access to such care remains uneven [1]. Dental disease can lead to pain, infection, and systemic complications and is a leading cause of missed school days [2]. Despite national preventive efforts, the burden of dental caries is strikingly concentrated: approximately 80% of caries in permanent teeth occur in just 25% of U.S. children, disproportionately affecting low-income and minority populations [3]. This inequity reflects more than individual behavior—it stems from structural barriers such as workforce shortages, geographic inaccessibility, and limited Medicaid participation [4,5]. These factors restrict timely treatment and perpetuate oral-health disparities.

Pediatric endodontic care offers a lens through which to view these systemic shortcomings. When deep caries approaches the pulp, many cases can be managed within general or pediatric dental practice, yet a substantial proportion of children are referred to endodontic specialists [6]. Such referrals, when not clinically necessary, delay care, increase costs, and strain specialty resources that are already scarce for publicly insured patients.

Nevada illustrates the challenge vividly. The state ranks near the bottom nationally in provider density and faces significant shortages of dental specialties, including endodontics [7]. These issues are particularly critical in pediatric endodontic care, which plays a vital role in preserving natural dentition and preventing premature tooth loss. A shortage of Medicaid-accepting endodontic specialists has resulted in reliance on university-based programs such as Advanced Education in General Dentistry (AEGD) clinics to treat underserved populations [8]. These clinics function both as safety-net providers and as training grounds that equip general dentists with advanced skills such as endodontic procedures. Analyzing referral patterns to these programs, therefore, provides a window into how multiple forces—diagnostic uncertainty, provider confidence, organizational policies, and financial pressures—may shape access to care.

Referral practices are central to the efficiency of this system. Evidence indicates that many dental referrals are avoidable, with patients presenting conditions well within the scope of primary care management [9]. Routine treatment for caries approaching a vital pulp, either by basic restorative fillings or vital pulp therapy (VPT), can often be performed by general or pediatric dentists. Yet unwarranted referrals persist, likely reflecting a combination of factors, including diagnostic and referral ambiguity, case complexity, limited experience with VPT, reimbursement policies, the absence of standardized pediatric referral protocols, Dental Service Organization (DSO) productivity pressures, and provider discomfort with behavioral management in younger children. Inappropriate referrals not only overburden specialty clinics but also delay treatment for patients with complex diseases who truly require specialist care [9].

These inefficiencies disproportionately affect underserved children, especially Medicaid beneficiaries [10]. Pediatric Medicaid patients often face additional barriers, including low provider participation, geographic isolation, and inadequate reimbursement [11]. These obstacles contribute to delays, added costs, and repeated diagnostic visits. As carious lesions progress over time, opportunities for successful conservative care diminish, increasing the likelihood for root canal therapy (RCT) when earlier VPT might have sufficed [12]. Such delays burden patients, families, and the healthcare system, especially when caregivers must travel long distances or miss work [13].

Improving diagnostic accuracy and strengthening primary-care management are potential avenues that may advance efficiency. VPT, encompassing pulp capping and pulpotomy, offers a conservative, cost-effective alternative to RCT for teeth with reversible pulpitis, supporting continued root development and lowering the risk of fractures over time [14,15,16]. Because outcomes worsen with delayed intervention, timely recognition and management of VPT-appropriate cases are essential [17].

Low Medicaid reimbursement rates, either overall or specific to endodontic procedures, may further limit provider participation, creating bottlenecks in specialty care [18]. As a result, few providers are incentivized to accept Medicaid, reducing timely care [10] and contributing to disease progression, pain, and tooth loss, thus undermining efforts to preserve oral health [19]. Furthermore, in settings with no established referral guidelines or where protocols are inconsistently implemented, dental practices may become financially or operationally motivated to refer cases externally rather than treating in the primary care setting.

Using electronic health record (EHR) data, this study evaluated the necessity of endodontic referrals to a University-based AEGD program and explored opportunities to enhance primary care delivery. Specifically, we analyzed the source, intent, and appropriateness of RCT referrals to determine how AEGD programs can reduce unnecessary specialty utilization and promote more equitable, patient-centered pediatric dental care.

## 2. Methods

A retrospective EHR review was conducted to analyze referral patterns and treatment outcomes for pediatric patients referred to two University-based AEGD clinics in Nevada. The Institutional Review Board determined the study to be non-human subject research.

The study included pediatric patients aged 6 to 17 years who were referred by external providers, including general dentists and pediatric dentists, for the evaluation and treatment of carious permanent teeth between February and August 2024. Primary teeth were excluded, as were trauma-related referrals and cases involving patients aged 18 years or older. Given the high volume of endodontic referrals received by the clinic, the selected time frame provided a representative sample of referral activity and patient characteristics. Although the authors recognize the clinical relevance e of molar incisor hypomineralization (MIH), this condition was not distinguished from caries-related lesions because of limited access to comprehensive dental histories. Incomplete documentation from referring providers prevented a reliable determination of lesion etiology, prior management, and progression.

Data collected included demographics (age, gender) and tooth-specific clinical data: tooth number and type (incisor, premolar, molar), pulpal diagnosis (healthy, reversible pulpitis, irreversible pulpitis, or necrotic), treatment recommendation (no treatment, filling only, VPT, RCT, or extraction), and treatment status (completed, scheduled, referred externally, or not completed).

Pulpal diagnosis of each tooth was determined following the guidelines of the American Academy of Pediatric Dentistry [20]. Teeth with no symptoms, palpation and percussion negative, tested normal to cold, demonstrated normal mobility, and with no radiographic pathology were diagnosed as having healthy pulps. Those teeth with elicited positive cold response of short duration, palpation and percussion negative, demonstrated normal mobility, and with no radiographic pathology were diagnosed as having reversible pulpitis. Teeth with reversible pulpitis can also have a history of intermittent symptoms that are short in duration to temperature and/or sweets, but are relieved with over-the-counter analgesics. Irreversible pulpitis is characterized by history or symptoms that are spontaneous, lingering, and not always responsive to pain medication. These teeth can also be accompanied by early signs of radiographic pathology, such as widened PDL, periapical radiolucency, internal/external root resorption, and furcation/periapical bone loss, along with mobility. Teeth with necrotic pulp would have no response to thermal or electric testing. Necrotic teeth frequently exhibit more advanced evidence of radiographic pathology and soft tissue pathology, such as fistula, sinus tract, swelling, and abscess, along with pain on percussion and/or palpation. History often includes significant pain with hallmarks of irreversible pulpitis that eventually went away when the pulp experienced necrosis.

Examinations were performed by AEGD residents under the supervision of experienced faculty, including a board-certified pediatric dentist. The availability of the pediatric dentist is critical, as level of cooperation can sometimes be difficult to achieve with younger patients in determining accurate diagnosis, as the history of pain, if there is any, is important. Accurate pulp diagnosis depends on integrating information from clinical examinations, radiographic findings, pulp testing, and the patient’s dental history. The clinician’s level of experience in behavioral guidance also plays a role in shaping the diagnostic outcome.

To promote diagnostic consistency, three faculty dentists independently reviewed and rated all cases. Each reviewer recorded their ratings without the knowledge of the other reviewers. To quantify agreement before discussion, inter-rater reliability was calculated using Fleiss’ kappa, which measures agreement among multiple raters beyond chance. Interpretation for kappa values of 0.61–0.80 indicates substantial agreement, and >0.80 indicates almost perfect agreement. Following independent ratings, all three reviewers participated in a structured consensus meeting, where any case with discrepant ratings was discussed in detail. Reviewers presented their rationale, and consensus was reached through deliberation until unanimous agreement was achieved. The consensus rating was then recorded as the final classification for the analysis, ensuring the highest possible accuracy of the final dataset.

Categorical variables were summarized as frequencies and percentages, and continuous variables as means with standard deviations or medians with interquartile ranges, as appropriate. Differences in patient age across the four pulpal-diagnosis categories (healthy, reversible pulpitis, irreversible pulpitis, and necrotic) were evaluated using the Kruskal–Wallis test because of non-normal distributions. Associations between gender and follow-up compliance (completed or scheduled treatment versus no follow-up) were assessed with chi-square tests; cases referred to outside providers were excluded from this comparison. Predictors of an appropriate referral—defined as a diagnosis of irreversible pulpitis or necrosis—were examined using multivariable logistic regression, including age, gender, Medicaid status, and clinic campus as covariates. Results were presented as adjusted odds ratios (OR) with 95% confidence interval (CI). All tests were two-sided with a significance level of α = 0.05, and analyses were performed using R version 4.3. All data were de-identified prior to analysis to ensure compliance with privacy regulations and IRB requirements.

## 3. Results

Between 1 February and 31 August 2024, a total of 154 children (mean age = 13.5 years) were referred for evaluation of permanent teeth requiring possible RCT. All referrals originated from private dental offices, most of which were affiliated with DSOs. The majority of patients (96.8%, n = 149) were enrolled in Medicaid. Female patients comprised 57.8% (n = 89) and male 42.2% (n = 65).

A total of 247 teeth were evaluated. Most referrals (66.9%, n = 103) involved a single tooth, while 33.1% (n = 51) involved two or more teeth. One patient was referred for eight teeth. The majority of referred teeth were molars (74.1%, n = 183), followed by anterior teeth (16.6%, n = 41) and premolars (9.3%, n = 23). Molars were referred approximately 4.5 times more often than anterior teeth and nearly 8 times more than premolars (Table 1).

Pulp vitality testing was completed for all 247 teeth. Diagnostic results indicated that 41.3% (n = 102) had healthy pulps, 16.6% (n = 41) had reversible pulpitis, 21.1% (n = 52) had irreversible pulpitis, and 20.2% (n = 50) had pulpal necrosis. Treatment recommendations included RCT for 37.2% (n = 92) of teeth, VPT for 37.7% (n = 93), restorative fillings for 13.0% (n = 32), and extraction for non-restorable teeth in 6.1% (n = 15). In 5.7% of cases (n = 14), no treatment was indicated (Table 1).

As of November 2024, 53.4% (n = 132) of referred teeth had completed treatment, while 26.3% (n = 65) had appointments scheduled. In 7.3% (n = 18), patients were referred back to the original referring office or redirected elsewhere. In 9.3% (n = 23), treatment was recommended, but patients did not schedule or attend follow-ups. (Table 1) Inter-rater reliability among the three faculty dentists was substantial, with Fleiss kappa values ranging from 0.680 to 1.000, indicating high diagnostic consistency.

A significant association was found between patient age and pulp status. Median age increased across diagnostic categories (Healthy: 12 years; Reversible: 14 years; Irreversible: 15 years; Necrotic: 14 years). A Kruskal–Wallis test confirmed a significant difference in age among the four pulpal-diagnosis groups (H = 15.86, *p* = 0.0012), indicating that older children were more likely to present with irreversible or necrotic pulp conditions (Table 2).

Gender was not significantly related to follow-up compliance. Among 220 evaluable cases (excluding external referrals), 197 children (89.5%) were compliant, meaning treatment was completed or scheduled. Compliance rates were similar for females (88%) and males (92%), with no statistically significant difference (χ^2^ = 0.52, *p* = 0.47) (Table 3).

In the multivariable logistic regression model, an “appropriate referral” was defined as a case diagnosed with irreversible pulpitis or necrosis. Age emerged as the only significant independent predictor: each additional year of age increased the odds of an appropriate referral by about 18% (adjusted OR = 1.18, 95% CI = 1.07–1.31, *p* = 0.0009) (Table 4). Gender, Medicaid enrollment, and clinic campus were not significantly associated with referral appropriateness. Overall, these findings indicate that older age was linked to greater diagnostic accuracy in referral decisions, while gender and insurance status had no measurable influence on compliance or referral appropriateness.

## 4. Discussion

This study analyzed the referral patterns from external general dentistry providers to the University-based AEGD clinics for pediatric dental care over a seven-month period. A total of 247 teeth from 154 children were included in the study, with pulpal diagnoses of healthy (41.3%), reversible pulpitis (16.6%), irreversible pulpitis (21.1%) and necrotic (20.2%).

The findings reveal significant inefficiencies in pediatric endodontic referral practices. More than half of the referred teeth (57.9%) were diagnosed as either healthy or exhibiting reversible pulpitis, conditions typically manageable in primary care settings. Children with these conditions might have received more timely and cost-effective care if treated within their dental home, avoiding unnecessary referrals to specialty clinics.

Additionally, age was significantly associated with pulp status, as older children were more likely to present with irreversible or necrotic pulp conditions.

This trend may indicate that disease severity may accumulate with delayed access to care, emphasizing the importance of earlier preventive and diagnostic interventions. Gender, by contrast, showed no relationship to follow-up compliance, indicating that missed appointments were likely driven by structural factors such as transportation or scheduling barriers rather than sex-based behavioral differences in adherence. Logistic regression confirmed that age was the only independent predictor of an appropriate referral, even after adjusting for gender, Medicaid status, and clinic campus, underscoring the need for early diagnosis and treatment to prevent disease progression and reduce avoidable specialty overuse.

### 4.1. Over-Referral and Diagnostic Challenges

The substantial proportion of referrals involving vital or minimally inflamed pulps suggests a trend of over-referral among general dentists. This places unnecessary demand on limited specialty resources, delays treatment for patients requiring complex care, and contributes to systemic inefficiencies [9,21]. For Medicaid-insured children already facing provider shortages, these delays can lead to pain, school absenteeism, financial strain, and disease advancement. The association between older age and appropriate referrals suggests that providers may exhibit diagnostic caution or behavioral apprehension when treating younger children, opting to refer even when cases fall within their scope. This pattern highlights the need for greater diagnostic confidence and behavioral management training in pediatric dental contexts.

Improving diagnostic accuracy among primary care dentists represents a key opportunity for system-level improvement. Enhanced predoctoral instruction in pulp testing and behavior management, along with continuing education focused on evidence-based diagnostic decision-making, may reduce uncertainty [21]. Furthermore, emerging technologies such as AI-supported diagnostic tools and teleconsultation platforms could help distinguish between vital and non-vital pulp conditions in ambiguous cases [22]. Implementing standardized diagnostic criteria and structured referral guidelines, supported by decision trees and flowcharts, would foster greater consistency and diagnostic reliability across practice settings.

### 4.2. VPT and Opportunities for Primary Care Management

In this study, VPT was recommended for 37.7% of referred teeth, representing a substantial subset suitable for conservative management. VPT—including indirect pulp capping, direct pulp capping, and pulpotomy—aims to preserve pulp vitality, promote root maturation, and reduce the risk of structural compromise in young permanent teeth [17]. Recent evidence even suggests that some cases of irreversible pulpitis may respond successfully to pulpotomy [20] expanding the opportunities for conservative treatment.

VPT is less invasive, more affordable, and less time-consuming than root canal therapy, making it an essential option for pediatric patients [17]. The finding that over half of the referred teeth were candidates for primary care management indicates an untapped potential to improve access and efficiency. Expanding provider training in VPT protocols and integrating contemporary pediatric-specific evidence-based guidelines into primary practice may reduce unnecessary specialty referrals and enhance quality [9].

Economic factors remain central. Low Medicaid reimbursement rates for time-intensive or complex procedures continue to discourage provider participation [23,24]. Pediatric Medicaid beneficiaries are thus more likely to be referred externally compared with privately insured children [24]. Fee schedules should be adjusted to reflect procedural complexity and time demands, ensuring that conservative care—although labor-intensive—is financially viable for primary providers.

Efforts to reduce inappropriate referrals should also focus on standardizing pediatric referral protocols. The development and implementation of evidence-based referral guidelines for pediatric endodontic care—supported by clinical algorithms and diagnostic checklists—can help reduce variability and improve the accuracy of referral decisions. In the long term, tools such as AI-assisted decision support systems may enhance diagnostic confidence and support more consistent treatment planning across providers. In addition, the lack of established referral guidelines or enforcement on a state and national level represents a systemic gap that contributes to variability in practice patterns. Without clear standards delineating which cases can be appropriately managed in primary care versus those requiring specialty referral, dentists, particularly those employed in large group practices or DSOs, may experience institutional pressure to refer rather than to treat. This very practice pattern was observed during the course of this study, where almost all patients were referred externally without initial caries control. Although the region’s Medicaid administrator expects the primary dentist to proceed with decay removal and possible temporization prior to referring [25]. Palliative treatment was not provided in the majority of patients in our study.

Setting standardized and enforceable referral criteria and aligning reimbursement policies with procedural complexity could collectively increase the capacity of general dentists to provide VPT, thereby improving timely access to care for children.

### 4.3. Barriers to Care Completion

This study identified logistical challenges that impacted care completion. Approximately 9.3% of patients failed to complete or schedule recommended treatment, reflecting persistent logistical and socioeconomic challenges. Non-completion was concentrated among Medicaid beneficiaries and likely related to systemic barriers such as provider shortages, transportation limitations, inflexible work schedules, and administrative burdens. These challenges compound delays in care and contribute to worsening oral health outcomes [26].

Addressing these barriers requires a multifaceted approach. Education outreach for caregivers can raise awareness of the importance of timely care [3], while policy reforms targeting transportation support, flexible scheduling, and reduced complexity are essential. Many families face broader structural constraints—limited childcare, language barriers, or reliance on public transit—that hinder consistent attendance.

While telehealth and virtual consultations could support future referral and follow-up systems, further research is needed to assess their feasibility in pediatric dentistry. Improving care continuity will require collaboration among general dentists, specialists, caregivers, and policymakers. Strengthening communication and follow-up coordination between providers may help mitigate non-adherence and promote equitable access. Ultimately, addressing the socioeconomic and structural roots of care discontinuity is critical to improving pediatric oral health outcomes.

### 4.4. Policy and Educational Implications

This study highlights the need for both educational and systemic policy change to improve pediatric dental care delivery. Dental schools should strengthen predoctoral curricula to include more comprehensive exposure to pediatric and endodontic cases, particularly emphasizing diagnostic reasoning, behavior management, and conservative treatment such as VPT [26]. Strengthening Commission on Dental Accreditation requirements to ensure direct clinical training with specialist faculty could enhance diagnostic accuracy and procedural competence. Postgraduate training programs such as AEGD and pediatric dental residencies can play a pivotal role in bridging the gap between education and clinical practice by providing continuing education and fostering collaboration with referring dental providers [8].

While improved training is one piece of the puzzle, enhancing Medicaid reimbursement and provider participation remains essential [18]. Low reimbursement fees for time-sensitive procedures have been widely acknowledged as one of the overarching drivers of high referral rates to university-based clinics [11]. Notably, some referrals in this study came from pediatric dentists who likely possessed the skills and knowledge for accurate diagnosis and completion of VPT procedures. Even with the implicit understanding of benefits associated with treating children in their routine pediatric dental home environment, these pediatric providers still referred externally. This observation, coupled with the scant participation of Medicaid-accepting endodontists, suggests that low reimbursement rates may be a central factor deterring treatment. Incentivizing provider participation through loan repayment, tax credits, and practice support programs may improve access in underserved areas [27]. Funding innovative care models, such as telehealth consultations, can facilitate collaboration between general dentists and specialists, reducing unnecessary referrals and ensuring timely treatment for complex cases [28]. Additionally, investment in data infrastructure and analytics expertise can help dental programs use real-world data to monitor referral patterns, assess outcomes, and guide ongoing quality improvement efforts.

In Nevada, Medicaid covers most dental services for children under the age of 21. Despite this coverage, reimbursement for procedures such as VPT and root canal treatment remains inadequate. Recently, several congressional committees have proposed a reduction in federal spending for Medicaid. These proposals include lowering the Federal Medical Assistance Percentage for states that expanded Medicaid under the Affordable Care Act and implementing per capita caps on federal matching dollars. Although these proposals are still under debate, such spending cuts could shift greater financial responsibility to individual states, forcing them to absorb a larger share of Medicaid costs to sustain enrollment and maintain service levels for beneficiaries [29].

Oral health is an integral component of overall health, yet it continues to receive less policy attention than medical insurance coverage. Numerous studies have demonstrated a bidirectional relationship between oral and systemic health. Periodontal disease, for example, has been associated with diabetes, metabolic syndrome, obesity, eating disorders, liver disease, cardiovascular disease, Alzheimer’s disease, rheumatoid arthritis, adverse pregnancy outcomes, and certain cancers [30]. Expanding Medicaid dental coverage and reimbursement is therefore not simply a fiscal consideration but a public-health imperative.

Persistently low reimbursement rates remain a principal reason many dentists decline to accept Medicaid or Medicare patients. Moreover, stagnant fees are prompting some current Medicaid providers to reconsider participation altogether [31]. According to the Nevada Medicaid Fee Schedule [32], payments for procedures such as direct and indirect pulp capping remain disproportionately low, and reimbursement for root canal treatments often fails to offset the costs of dental materials, instrumentation, and chair time. These financial constraints encourage over-referral or inconsistent referral patterns and contribute to the shortage of Medicaid-accepting specialists—particularly endodontists.

Referrals are an essential aspect of dental practice; however, criteria guiding referral decisions are often inconsistently defined or poorly standardized. Dental education emphasizes that referral choices depend on a provider’s knowledge, clinical experience, and comfort with specific procedures. No single dentist can perform every type of care, yet appropriate referral requires clear, ongoing communication between providers and patients to ensure continuity of treatment [33]. For example, pediatric dentists possess advanced behavioral-management skills that general dentists may lack. Young children often struggle with cooperation, experience anxiety, or lack cognitive understanding of treatment procedures. Among children and adolescents, approximately 9% experience dental anxiety severe enough to impede care [34].

DSOs have become increasingly prevalent, owning and managing multiple practices while providing administrative, marketing, and operational support. While DSOs can enhance efficiency, concerns persist regarding their potential influence on care quality and provider autonomy. The number of DSOs in the United States has expanded from roughly 100 in 2010 to over 2000 in 2023, with an estimated compound annual growth rate of 15.8% projected through 2030 [33]. This growth reflects rising demand for dental services, reduced dentist ownership interest, and an aging population. Although DSOs provide organizational stability, they may also require higher patient throughput, longer work hours, and productivity targets, potentially contributing to burnout and diminished job satisfaction. When DSOs accept Medicaid, these productivity expectations can influence clinical decision-making, including whether providers elect to complete VPT procedures versus referring patients externally.

Accurate diagnosis remains fundamental to high-quality dental care. Clinicians must systematically synthesize patient history, clinical findings, and radiographic evidence to establish a reliable diagnosis. Medical and dental histories should inform diagnostic hypotheses, which are then verified through clinical examination, periodontal evaluation, and pulp testing. In some cases, diagnostic findings are ambiguous or conflicting, making it impossible to confirm pulpal status. In such situations, deferring treatment and re-evaluating the patient—or seeking specialist consultation—is the most appropriate course of action [35].

Medicaid coverage varies widely by state, as federal law does not mandate comprehensive adult dental benefits. Consequently, each state determines its own coverage scope. While many states have expanded adult dental benefits in recent years, significant disparities persist. For example, New York and Oregon provide comprehensive dental benefits for adults, whereas Nevada and similar states restrict coverage primarily to emergency services [36].

This study focuses on children aged 6–17 years, a population covered under the Early and Periodic Screening, Diagnostic, and Treatment benefit. Under federal law, states must provide all necessary dental services to maintain oral health, relieve pain, and restore function [37]. However, the scope and frequency of preventive and restorative services vary substantially across states, depending on local Medicaid policy and funding [38].

According to a Health Policy Institute survey, dentists identified low reimbursement, limited procedural coverage, and administrative burdens—including prior authorization requirements and complex credentialing major deterrents to Medicaid participation [39]. Dental practice structure may mitigate some of these barriers: dentists affiliated with DSOs tend to report higher Medicaid participation rates than solo practitioners, likely due to economies of scale and centralized administrative systems [37].

Expanding state-funded facilities and publicly supported institutions, including community health centers, dental schools, and post-doctoral education programs, would improve access for underserved children while serving as essential training environments. Legislative support for these initiatives, combined with comprehensive reimbursement reform, could reduce inappropriate referrals and foster more equitable pediatric dental care delivery.

Overall, these findings underscore the need to align Medicaid reimbursement with evidence-based clinical practice. The observed age-related differences in referral appropriateness and the high rate of potentially avoidable referrals suggest that financial incentives, provider training, and diagnostic support tools should be systematically integrated into Medicaid policy and broader public-health strategies. Policies that reward timely, conservative care, including enhanced reimbursement for VPT, teleconsultation support with specialists, and inclusion of diagnostic quality metrics in Medicaid performance evaluations, could reduce unnecessary specialty referrals and improve equitable access to pediatric endodontic care.

### 4.5. Limitations and Future Research

This study was limited to two University-affiliated AEGD clinics in Nevada, which may restrict the generalizability of findings to other regions or healthcare systems. Because Nevada’s provider landscape and Medicaid reimbursement rates differ substantially from those of other states, the results should be interpreted with caution when applied elsewhere. In particular, variations in provider availability, state-level Medicaid coverage policies, and participation incentives may lead to different referral patterns and treatment outcomes in other geographic or organizational contexts. State-level differences in Medicaid policies, reimbursement structures, and provider availability could influence both referral behaviors and treatment outcomes. Future research incorporating multi-institutional and multi-state data would provide a more comprehensive understanding of referral trends and contextual factors shaping pediatric endodontic care.

Additionally, primary teeth were excluded because they do not require root canal therapy and are typically managed effectively within pediatric dental practice. Trauma-related cases were also excluded to maintain the study’s focus on disease burden related to caries and delayed preventive or interventive care. Teeth affected by MIH were not separately identified, which limited the ability to distinguish caries of developmental origin from typical carious lesions. This limitation reflects the AEGD clinics’ specialized focus on endodontic referrals rather than comprehensive dental assessment. Primary care providers retained responsibility for risk assessment, preventive management, and early intervention, while the AEGD clinic’s role was restricted primarily to endodontic treatment.

When diagnoses of healthy pulp or reversible pulpitis were established, VPT was performed instead of RCT as a more conservative and biologically sound option. The goal of VPT and other conservative interventions is to preserve the natural tooth structure for as long as possible. Patients were reminded that each additional invasive procedure increases the risk of eventual tooth loss. Given that the average lifespan of an RCT-treated tooth is approximately 10–20 years—shorter if a permanent restoration is delayed—deferring RCT when clinically appropriate is in the best interest of pediatric patients to maintain natural dentition.

Provider-level data were not collected, preventing assessment of potential differences in diagnostic accuracy between AEGD residents and supervising faculty. Only one board-certified pediatric dentist participated in clinical evaluations. The remainder of assessments were performed by three general dentistry residents (each with 0–1 years of post-graduate experience) and three general dentistry faculty members with 5 to15 years of experience across settings such as predoctoral education, hospital dentistry, care for special health needs, traditional private practice, and implant-focused practice. Most cases were assessed by residents, but exact proportions could not be determined without re-reviewing the complete dataset.

Future studies should incorporate provider-level variables, such as training, experience, and practice model, to enable more robust statistical analyses of diagnostic accuracy. Such data could reveal differences between residents and faculty and identify whether years of experience correlate with greater diagnostic precision or more conservative treatment planning.

This study also did not capture patient-level factors, such as caregiver education, socioeconomic status or clinic distance, which may affect access to and completion of care. Integrating these variables in future research, along with qualitative interviews of patients and providers, could yield deeper insights into referral barriers, systemic inequities, and training needs. Long-term, prospective studies evaluating VPT success rates in pediatric populations are also needed to determine the durability and cost-effectiveness of conservative pulp therapies compared with RCT.

## 5. Conclusions

This study reveals substantial inefficiencies in pediatric endodontic referral practices, with more than half of referred teeth suitable for management within primary care. Older patient age emerged as the only independent predictor of an appropriate referral, suggesting that delays in routine or preventive care contribute to greater disease severity at presentation. Gender and insurance status showed no significant association with follow-up compliance or referral appropriateness.

To address these issues, efforts should focus on strengthening diagnostic training, standardizing referral criteria, and incentivizing provider participation in Medicaid programs. Equally important are systemic policy reforms that improve reimbursement for conservative procedures, reward timely VPT, and promote integrated referral protocols. Collectively, these strategies can reduce unnecessary specialty referrals, enhance diagnostic accuracy, and promote more equitable, efficient, and patient-centered pediatric dental care.

## Figures and Tables

**Table 1:** Teeth Distribution, Pulp Condition, and Treatment Status of Referred Teeth.

Variable	Number	Percentage
Teeth type		
Anterior	41	16.6%
Premolar	23	9.3%
Molar	183	74.1%
Pulp condition		
Healthy Pulps	102	41.3%
Reversible Pulpitis	41	16.6%
Irreversible Pulpitis	52	21.1%
Pulp Necrosis	50	20.2%
Treatment status		
Completed	132	53.4%
Scheduled	65	26.3%
Referred to Other	18	7.3%
No Follow Up	23	9.3%

**Table 2:** Age by Pulp Status.

Pulp Status	N	Mean Age (SD)	Median (IQR)
Healthy	102	12.6 (2.8)	12 (11–15)
Reversible	41	13.6 (2.9)	14 (11–16)
Irreversible	52	14.4 (2.3)	15 (13–16)
Necrotic	50	13.8 (2.7)	14 (12–17)

**Table 3:** Gender versus Follow-up Compliance.

Gender	Compliant n (5)	No Follow-Up n (%)
Female	117 (88.0)	16 (12.0)
Male	80 (92.0)	7 (8.0)

**Table 4:** Logistic Regression Predicting Appropriate Referral of Irreversible Pulpitis or Necrosis.

Predictor	Odds Ratio	95% CI	*p*-Value
Age (per year)	1.18	1.07–1.31	0.0009
Male (versus Female)	0.78	0.46–1.33	0.3687
Medicaid (yes versus no)	0.82	0.23–2.91	0.7527
Campus: Summerlin (versus Henderson)	1.21	0.70–2.09	0.4937

## Data Availability

The data presented in this study are available on request from the corresponding author with IRB approval.

## References

[1] Hung M., Moffat R., Gill G., Lauren E., Ruiz-Negrón B., Rosales M.N., Richey J., Licari F.W. (2019). Oral health as a gateway to overall health and well-being: Surveillance of the geriatric population in the United States. Spec. Care Dentist.

[2] Agaku I.T., Olutola B.G., Adisa A.O., Obadan E.M., Vardavas C.I. (2015). Association between unmet dental needs and school absenteeism because of illness or injury among U.S. school children and adolescents aged 6–17 years, 2011–2012. Prev. Med..

[3] CMS Guide to Children’s Dental Care in Medicaid. Departement of Health and Human Services, Ocotober 2004. https://www.medicaid.gov/medicaid/benefits/downloads/child-dental-guide.pdf.

[4] Ma S., Serban N., Dehghanian A., Tomar S.L. (2023). The impact of dentists’ availability in delivering dental care in Florida Elementary Schools. J. Public Health Dent..

[5] Richardson S.L., Khan A.A., Rivera E.M., Phillips C. (2014). Access to endodontic care in North Carolina public health and Medicaid settings. J. Public Health Dent..

[6] Endodontists A.A.o. Why See An Endodontist. American Association of Endodontists: 2013. https://www.aae.org/patients/why-see-an-endodontist/.

[7] Medicaid N. Nevada Medicaid and Nevada Check Up Provider Web Portal. Nevada Department of Health and Human Services. https://www.medicaid.nv.gov/.

[8] Spears R., Leite L.P., Schnell R.A., Dellinges M., Brooks H.E., Itaya L.E. (2013). AEGD programs: Why now, why more?. J. Dent. Educ..

[9] Burke F.J., Goodall C.A., Hayes F. (1999). Appropriate and inappropriate referrals to a unit of conservative dentistry. Prim. Dent. Care.

[10] Mofidi M., Rozier R.G., King R.S. (2002). Problems With Access to Dental Care for Medicaid-Insured Children: What Caregivers Think. Am. J. Public Health.

[11] Chalmers N.I., Compton R.D. (2017). Children’s Access to Dental Care Affected by Reimbursement Rates, Dentist Density, and Dentist Participation in Medicaid. Am. J. Public Health.

[12] Bjørndal L. (2008). The caries process and its effect on the pulp: The science is changing and so is our understanding. Pediatr. Dent..

[13] Bupa (2023). Employees Delay Dental Treatment Due to Time off Work Worries.

[14] Schwendicke F., Stolpe M. (2014). Direct pulp capping after a carious exposure versus root canal treatment: A cost-effectiveness analysis. J. Endod..

[15] Wells C., Dulong C., McCormack S. (2019). CADTH Rapid Response Reports. Vital Pulp Therapy for Endodontic Treatment of Mature Teeth: A Review of Clinical Effectiveness, Cost-Effectiveness, and Guidelines.

[16] Zhang W., Yelick P.C. (2010). Vital pulp therapy-current progress of dental pulp regeneration and revascularization. Int. J. Dent..

[17] Schwendicke F., Herbst S.R. (2023). Health economic evaluation of endodontic therapies. Int. Endod. J..

[18] Buchmueller T.C., Levinson Z.M., Levy H.G., Wolfe B.L. (2016). Effect of the Affordable Care Act on Racial and Ethnic Disparities in Health Insurance Coverage. Am. J. Public Health.

[19] Kaushik M., Sood S. (2023). A Systematic Review of Parents’ Knowledge of Children’s Oral Health. Cureus.

[20] American Academy of Pediatric Dentistry (2021). Pulp therapy for primary and immature permanent teeth. The Reference Manual of Pediatric Dentistry.

[21] Coppola N., Baldares S., Blasi A., Bucci R., Spagnuolo G., Mignogna M.D., Leuci S. (2021). Referral Patterns in Oral Medicine: A Retrospective Analysis of an Oral Medicine University Center in Southern Italy. Int. J. Environ. Res. Public Health.

[22] Hung M., Xu J., Lauren E., Voss M.W., Rosales M.N., Su W., Ruiz-Negrón B., He Y., Li W., Licari F.W. (2019). Development of a recommender system for dental care using machine learning. SN Appl. Sci..

[23] Burns L.E., Gencerliler N., Ribitzki U., Yashpal S., Feldman L., Sigurdsson A., Gold H.T. (2024). Access to Care Considerations for the Endodontic Treatment of Immature Permanent Teeth: A National Survey of Pediatric Dentists and Endodontists. J. Endod..

[24] McQuistan M.R., Kuthy R.A., Daminano P.C., Ward M.M. (2006). General dentists’ referrals of 3- to 5-year-old children to pediatric dentists. J. Am. Dent. Assoc..

[25] Liberty Dental Plan Nevada Provider Reference Guide. https://www.libertydentalplan.com/Resources/Documents/Nevada%20Provider%20Reference%20Guide.pdf.

[26] Benjamin R.M. (2010). Oral Health: The Silent Epidemic. Public Health Rep..

[27] Casamassimo P.S., Seale N.S., Rutkauskas Ii J.S., Rutkauskas J.S. (2018). Are U.S. Dentists Adequately Trained to Care for Children?. Pediatr. Dent..

[28] Arevalo O., Sears L., Saman D.M., Roldan R. (2020). Evaluation of Potential Strategies to Increase Provider Participation in Florida’s Dental Medicaid Program. Pediatr. Dent..

[29] Williams E., Rudowitz R., Burns A., Euhus R. A Medicaid Per Capita Cap on the ACA Expansion Population: State by State Estimates. https://www.kff.org/medicaid/a-medicaid-per-capita-cap-on-the-aca-expansion-population-state-by-state-estimates/.

[30] Kapila Y.L. (2021). Oral health’s inextricable connection to systemic health: Special populations bring to bear multimodal relationships and factors connecting periodontal disease to systemic diseases and conditions. Periodontology 2000.

[31] Cortigiano C. Why Dentists Do and Don’t Accept Medicaid. https://www.beckersdental.com/featured-perspectives/why-dentists-do-and-dont-accept-medicaid/.

[32] FEE SCHEDULES NV.gov. https://dhcfp.nv.gov/resources/rates/feeschedules/.

[33] DDSmatch The Rise of DSOs in Dentistry: The Pros and Cons of Joining a Dental Service Organization. NDDS. https://www.ndds.org/advocacy/legislative-insider/2025/04/11/the-rise-of-dsos-in-dentistry--the-pros-and-cons-of-joining-a-dental-service-organization.

[34] ADA Specialty Referrals: Guidelines for Practice Success|Managing Patients|Policies. American Dental Association. https://www.ada.org/resources/practice/practice-management/specialty-referrals.

[35] AAE Endodontics: Colleagues for Excellence. Endodontic Diagnosis. https://www.aae.org/specialty/wp-content/uploads/sites/2/2017/07/endodonticdiagnosisfall2013.pdf.

[36] CHCS Medicaid Adult Dental Benefits Coverage by State. Septemeber 2019. https://www.chcs.org/media/Medicaid-Adult-Dental-Benefits-Overview-Appendix_091519.pdf.

[37] Does Medicaid Cover Dental Care? U.S. Department of Health and Human Services. https://www.hhs.gov/answers/medicare-and-medicaid/does-medicaid-cover-dental-care/index.html.

[38] Medicaid Dental Care. Medicaid. https://www.medicaid.gov/medicaid/benefits/dental-care.

[39] Versaci M.B. ADA Calls for Policy Reforms to Improve Medicaid Access. American Dental Association. https://adanews.ada.org/ada-news/2024/august/ada-calls-for-policy-reforms-to-improve-medicaid-access/.

